# Alveolar Abscess.—Treatment with Carvacrol

**Published:** 1875-05

**Authors:** H. L. Sage


### ALVEOLAR ABSCESS.—TREATMENT WITH CARVACROL.

BY H. L. SAGE, D. D. S.

In my report on Dental Therapeutics, published in the Transactions of the American Dental Association, I introduced some statements in regard to cases of alveolar abscess in which carvacrol alone was employed in their treatment with success. I wish now to report several cases in addition, and at some leisure moment may refer, by some cases in practice, to its virtues in other directions.

Mr. J. C., age about sixty, sanguine temperament; called Nov. 12th, 1873, with a large swelling over the right first superior bicuspid.

This tooth was worn down by a pipe stem, and the pulp finally died in consequence, though the irritation produced by the abrasion had kept it sealed up by a deposit of secondary dentine.

First step: lanced the gum and emptied the swelling of pus. Next, opened and cleansed the pulp cavity, which was not so large as in softer teeth, this tooth being of the hard dense, yellow kind. Pumped carvacrol into the root, but it failed to appear through the gum. Drilled through the alveolus without difficulty and with scarcely any pain, striking the apex of the root.

Carried carvacroled silk (silk saturated with carvacrol) through the opening in the gum and alveolus into the abscess, leaving the end protruding, with instructions to the patient to remove it before morning if much swelling followed. (He pulled out the silk on the following day.) Filled the root temporarily with carvacroled floss silk. The carvacrol caused no smarting or burning sensation in this case, probably on account of the presence of much pus on the secreting surface of the abscess. (?) After an interval of two days Mr. C.

again appeared. Some pus present, but swelling reduced. Injected carvacrol into the abscess through the fistula.

May 2d, 1873, saw the case for the first time since making the last application. Found the gum over the tooth hard and perfectly healthy, the fistula closed, and everything indicating the entire removal of the disease. The parts have been entirely comfortable ever since.

W. S. H., a young man of bilious temperament, called May 5, 1873, with the right superior lateral incisor dead. Removed pulp and cleansed the root. Pumped in carvacrol; no sensation. This indicated that the medicines did not pass through the nerve canal into the socket, though not positive proof, as the treatment of some recent cases has shown. Saturated floss silk with carvacrol and filled the root therewith, closing the crown cavity with oxychloride of zinc. No trouble occurred. After ten days, removed the temporary filling and filled the root and crown cavity with gold.

May 15th: Mr. H. again called.

Left superior lateral incisor, dead and offensive. Drilled into the palatine surface, removed decomposed portions of pulp, and pumped in carvacrol, which was followed by the burning, peppery sensation in the socket, at the end of the root. There had been no pulp exposure in this tooth, to cause death, though just previously, the pulp then being dead, I had filled a cavity in the anterior proximal surface. Filled pulp cavity with carvacroled floss silk, and the orifice with oxychloride.

In three days the patient came in with face swollen over the tooth. Lanced the gum at the apex of the root and drilled through the alveolus striking the root. Not being able to proceed with the treatment, on account of the hemorrhage, placed a cotton pellet saturated with tinct. iodine in the orifice of the fistula, to keep it from closing, but he removed it in the course of an hour.

Next day the swelling having increased, prescribed a dressing of cold water, stimulated with alcohol, vinum opii or brandy. Under this treatment the inflammation rapidly subsided. Then treated the abscess with carvacrol until a cure was affected. Subsequently filled the root and crown cavity with gold.

A. S. T., lawyer, age about fifty. Tooth, left first superior bicuspid, abscessed, with fistula in the gum, discharging thick, cheesy, curd-like pus.

Chronic case; two roots. Treated by pumping carvacrol through the buccal root, which appeared through the fistula, causing a very severe burning sensation in the socket and flushing the face.

This treatment was given May 12, 1873, and the root and crown temporarily filled as in the cases before cited. Probably it was repeated at a subsequent sitting.

February 17, 1874, the temporary filling was removed and gold substituted. The fistula was found to be closed by hard cicatricial tissue and the parts healthy.

Mrs. S. G., middle aged lady, in good condition called February 4, 1874. Tooth, left 2d superior bicuspid, with a root filling of cotton and a crown filling of Hill's stopping.

Soreness over the root at the apex; no fistula. Cleaned out the root and pumped in carvacrol which did not seem to pass through the apicial foramen. Filled temporarily as in the cases before repeated, saturating the silk with carvacrol. When the patient again appeared (at her convenience) the soreness was but slight.

Repeated the treatment two or three times according to convenience, when the tooth was ready for the reception of a permanent filling.

The foregoing cases, from among others, are cited not because they present phases different from those which ordinarily appear, but to show that carvacrol has been and may be made effectual in the treatment of alveolar abscess.

More than two years since, the writer successfully employed this agent in the treatment of a chronic case of seventeen years standing, and was probably the first to practically demonstrate its value in this direction. Other agents, doubtless, may be and are more suitable in certain cases. I make use of them when the indications warrant.

Carvacrol, being possessed of mild caustic or escharotic properties, may be less rapid in changing a pus secreting to a "plasm producing" surface, than in cases where more caustic agents are required; at the same time it is less irritating and therefore may be safer, from the fact that the danger from over medication is less imminent. Indeed it seems many times to reduce inflammation.

Carvacrol may be more potent in some other directions than in the treatment of alveolar abscess; for instance, as a sedative to exposed pulps, as an obtunder of sensitive dentine for which it is pre-eminently adapted, and stands without an, equal. It will penetrate sound dentine almost immediately, as is evidenced by the fact that it will often remove the sensitiveness from the abraded surfaces of teeth in from one to two minutes, whether the abrasion is spontaneous or mechanical —a statement admitting of positive proof. But it is not intended to enlarge upon these points at present.

